# Supplementary material for: Blockade of PGK1 and ALDOA enhances bilirubin control of Th17 cells in Crohn’s disease
Source: Commun Biol. 2022 Sep 21;5:994. doi: 10.1038/s42003-022-03913-9 (PMC9492699; doi:10.1038/s42003-022-03913-9)
Supplement: Supplementary file 2 — Supplementary Information [file 42003_2022_3913_MOESM2_ESM.pdf]

Blockade of *PGK1* and *ALDOA* enhances bilirubin control of Th17 cells in Crohn's disease

Marta Vuerich, Na Wang, Jonathon J. Graham, Li Gao, Wei Zhang, Ahmadreza Kalbasi, Lina Zhang, Eva Csizmadia, Jason Hristopoulos, Yun Ma, Efi Kokkotou, Adam S. Cheifetz, Simon C. Robson, Maria Serena Longhi

Supplementary Material

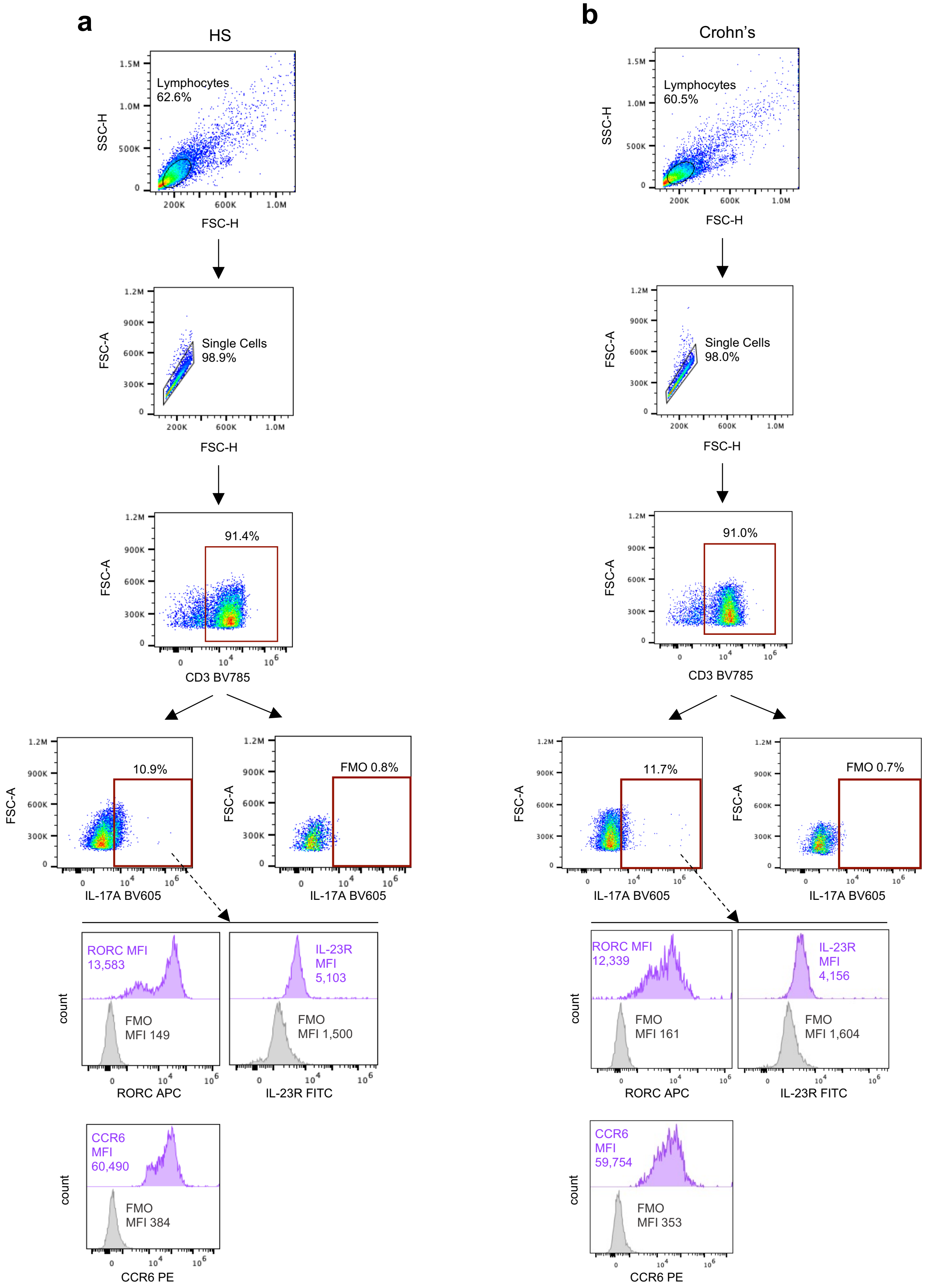

Supplementary Fig. 1: Frequency of IL-17 producing cells after CD4 cell polarization.

CD4 cells were isolated from the peripheral blood of healthy subjects and patients with Crohn's disease and exposed to IL-6, IL-1β and TGF-β for five days. The frequency of IL-17A producing lymphocytes in the polarized cells was measured by flow cytometry. Cells were initially gated on live lymphocytes and subsequently on single cells and CD3 lymphocytes. Expression of the Th17 cell markers RORC, IL-23R and CCR6 was measured in the $CD3^{+}IL\text{-}17^{+}$ subset. Representative flow cytometry plots of FSC-H and SSC-H, FS-H and FS-A, CD3 Brilliant Violet 785 (BV785) and FSC-A, and IL-17A BV605 and FSC-A from one healthy subject and one patient with Crohn's disease are shown. Histograms of RORC APC, IL-23R FITC and CCR6 PE are also shown. Cell frequencies and mean fluorescence intensity (MFI) are indicated in each plot or histogram. Gates were drawn based on fluorescence minus one (FMO) control staining.

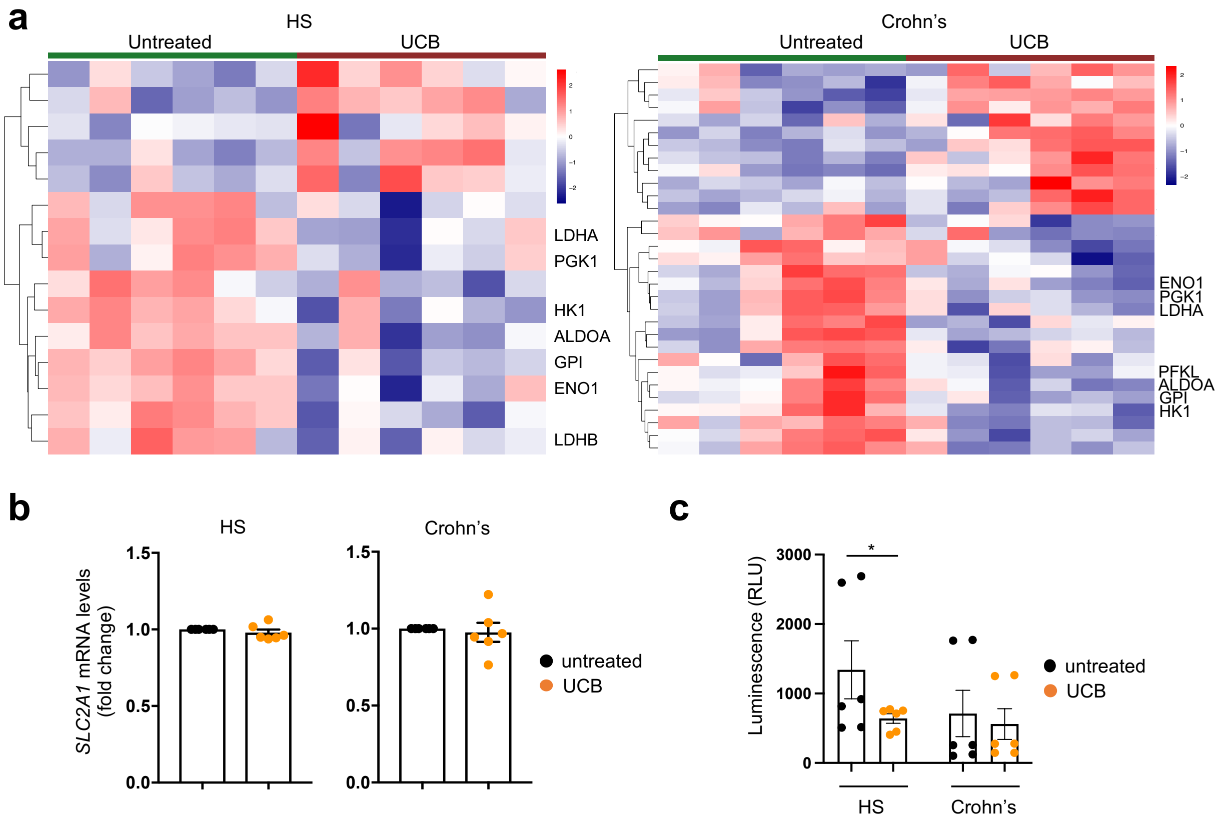

Supplementary Fig. 2. Effects of unconjugated bilirubin on Th17 cell metabolism.
(a) Heatmaps representing the differentially expressed genes (DEGs) across the metabolic pathways included in the nCounter Vantage RNA Cancer Metabolism NanoString panel between unconjugated bilirubin (UCB) treated and untreated Th17 cells of healthy subjects (HS, n=6) and Crohn's disease patients (n=6). DEGs were defined based on P≤0.05 (false discovery rate ≤0.05 in all cases). (b) Mean±SEM *SLC2A1* mRNA levels (obtained by NanoString) in untreated and UCB treated Th17 cells obtained from the peripheral blood of healthy subjects (HS, n=6) and Crohn's disease patients (n=6). (c) Uptake of glucose by untreated and UCB treated Th17 cells was determined using the Glucose Uptake-Glo Assay. Mean±SEM relative light unit (RLU) is shown for Th17 cells obtained from n=6 HS and n=6 patients with Crohn's disease. *P≤0.05 using two-sided paired *t* test.

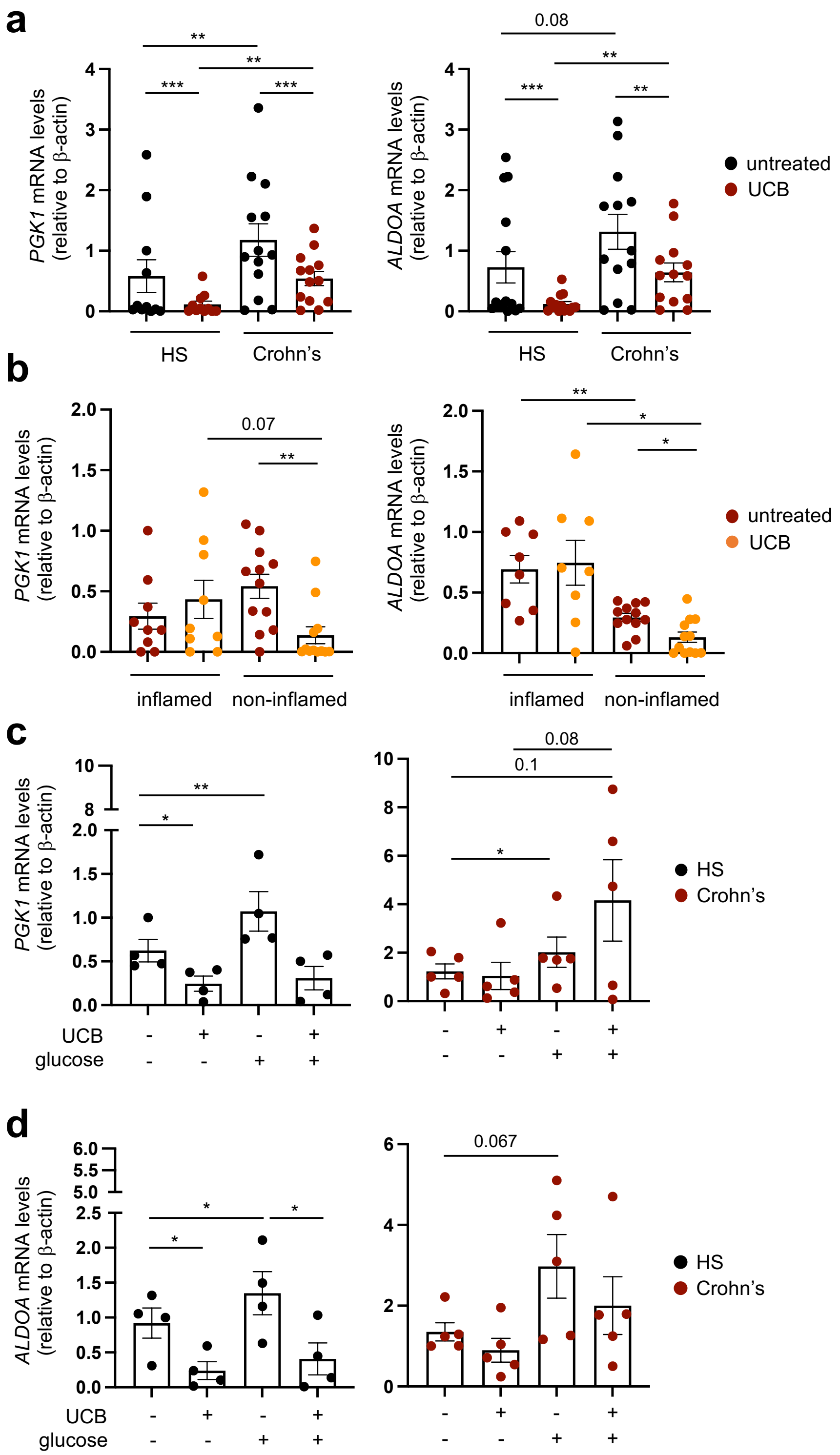

Supplementary Fig. 3. Effects of unconjugated bilirubin on Th17 cell *PGK1* and *ALDOA* expression.

Mean±SEM *PGK1* and *ALDOA* mRNA levels in untreated and unconjugated bilirubin (UCB) treated Th17 cells obtained from (a) the peripheral blood of healthy subjects (n=11 for *PGK1* and n=13 for *ALDOA*) and Crohn's disease patients (n=13 for *PGK1* and n=12 for *ALDOA*) and from

(b) inflamed (n=9 for *PGK1* and n=8 for *ALDOA*) and non-inflamed (n=12) biopsied areas of Crohn's disease patients. Mean±SEM (c) *PGK1* and (d) *ALDOA* mRNA levels in Th17 cells, untreated or exposed to UCB, glucose and UCB plus glucose (n=4 HS and n=5 Crohn's disease patients). *P≤0.05, **P≤0.01 and ***P≤0.001 using one-way ANOVA test, followed by Tukey's multiple comparison test.

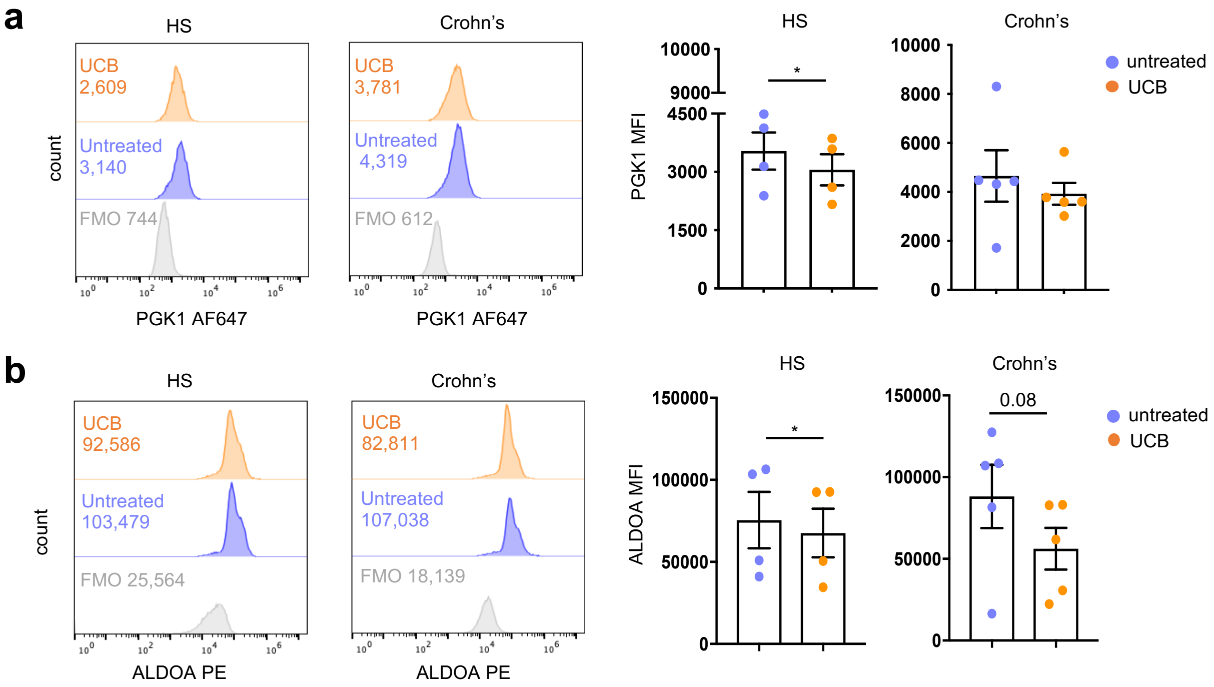

Supplementary Fig. 4. Effects of unconjugated bilirubin on PGK1 and ALDOA levels in health and Crohn's disease.

(a-b) Histograms of PGK1 Alexa Fluor 647 (AF647) and ALDOA PE fluorescence in untreated and unconjugated bilirubin (UCB) treated Th17 cells from one representative healthy subject (HS) and one patient with Crohn's disease. Values of mean fluorescence intensity (MFI) are indicated within each histogram. PGK1 and ALDOA positivity was established based on fluorescence minus one (FMO) control staining. Mean±SEM PGK1 and ALDOA MFI in untreated and UCB treated Th17 cells from HS (n=4) and patients with Crohn's disease (n=5). *P≤0.05 using two-sided paired *t* test.

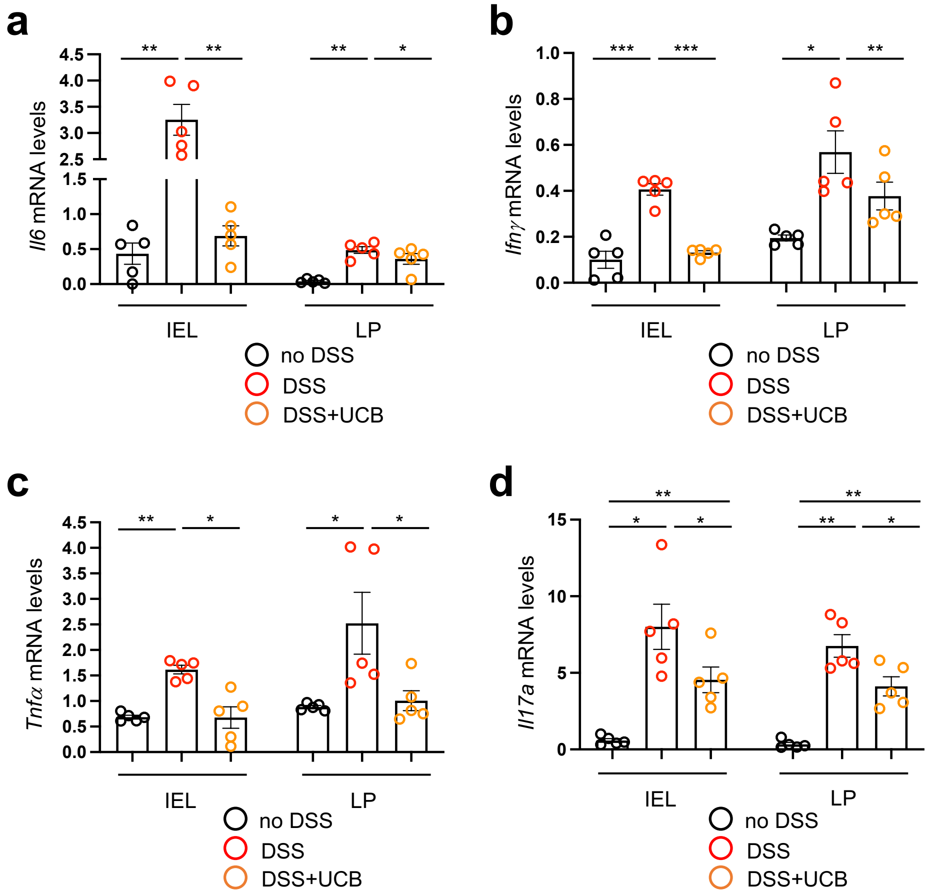

Supplementary Fig. 5. Levels of proinflammatory cytokines in intra-epithelial and lamina propria CD4 lymphocytes in mice with DSS colitis.

Levels of *Il6*, *IFNγ*, *Tnfα* and *Il17a* proinflammatory cytokines were measured by qPCR in CD4 cells isolated from intra-epithelial (IEL) and lamina propria (LP) lymphocytes of wild type mice subjected to vehicle, dextran sulfate sodium (DSS) or DSS and unconjugated bilirubin (UCB). Mean±SEM (a) *Il6*, (b) *IFNγ*, (c) *Tnfα* and (d) *Il17a* levels in IEL and LP derived CD4 cells (vehicle n=5; DSS n=5; DSS+UCB=5). CD4 cells from the IEL and LP compartments of DSS mice display heightened *Il6*, *Ifnγ*, *Tnfα* and *Il17a* levels, when compared to CD4 cells obtained from mice exposed to vehicle (n=5). Treatment with UCB reduces the levels of these pro-inflammatory cytokines in both compartments. *P≤0.05, **P≤0.01 and ***P≤0.001 one-way ANOVA test, followed by Tukey's multiple comparison test.

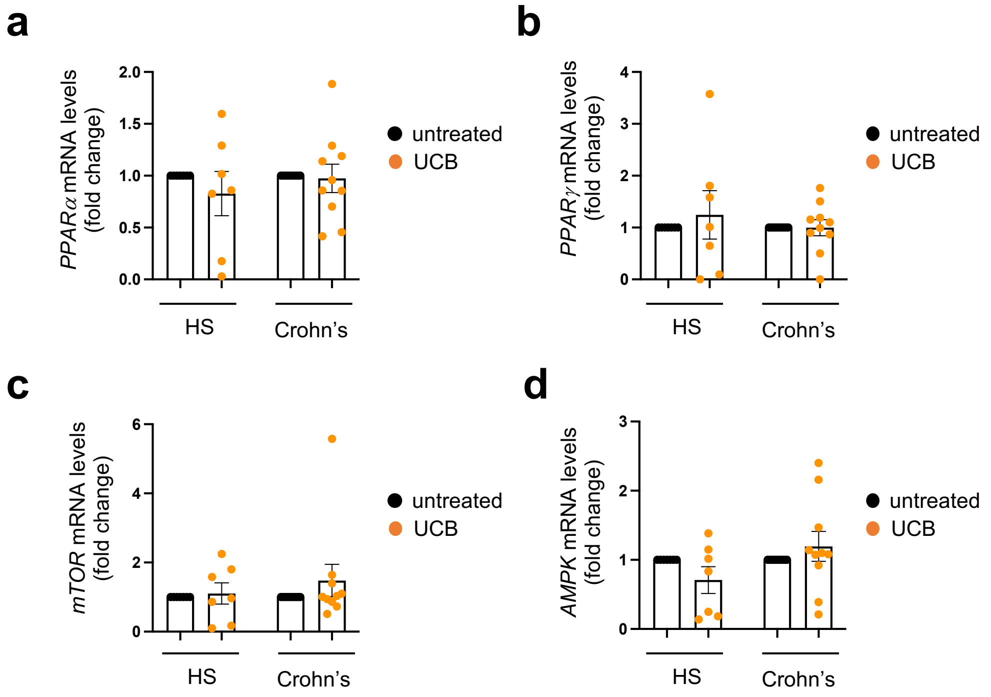

Supplementary Fig. 6. Effects of unconjugated bilirubin on metabolic genes.
Levels of *PPARα*, *PPARγ*, *mTOR* and *AMPK* were measured by qPCR in untreated and unconjugated bilirubin (UCB) treated Th17 cells of HS and patients with Crohn's disease. Mean±SEM (a) *PPARα*, (b) *PPARγ*, (c) *mTOR* and (d) *AMPK* mRNA levels in untreated and UCB treated Th17 cells of HS *(*n=7) and patients with Crohn's disease (n=10).

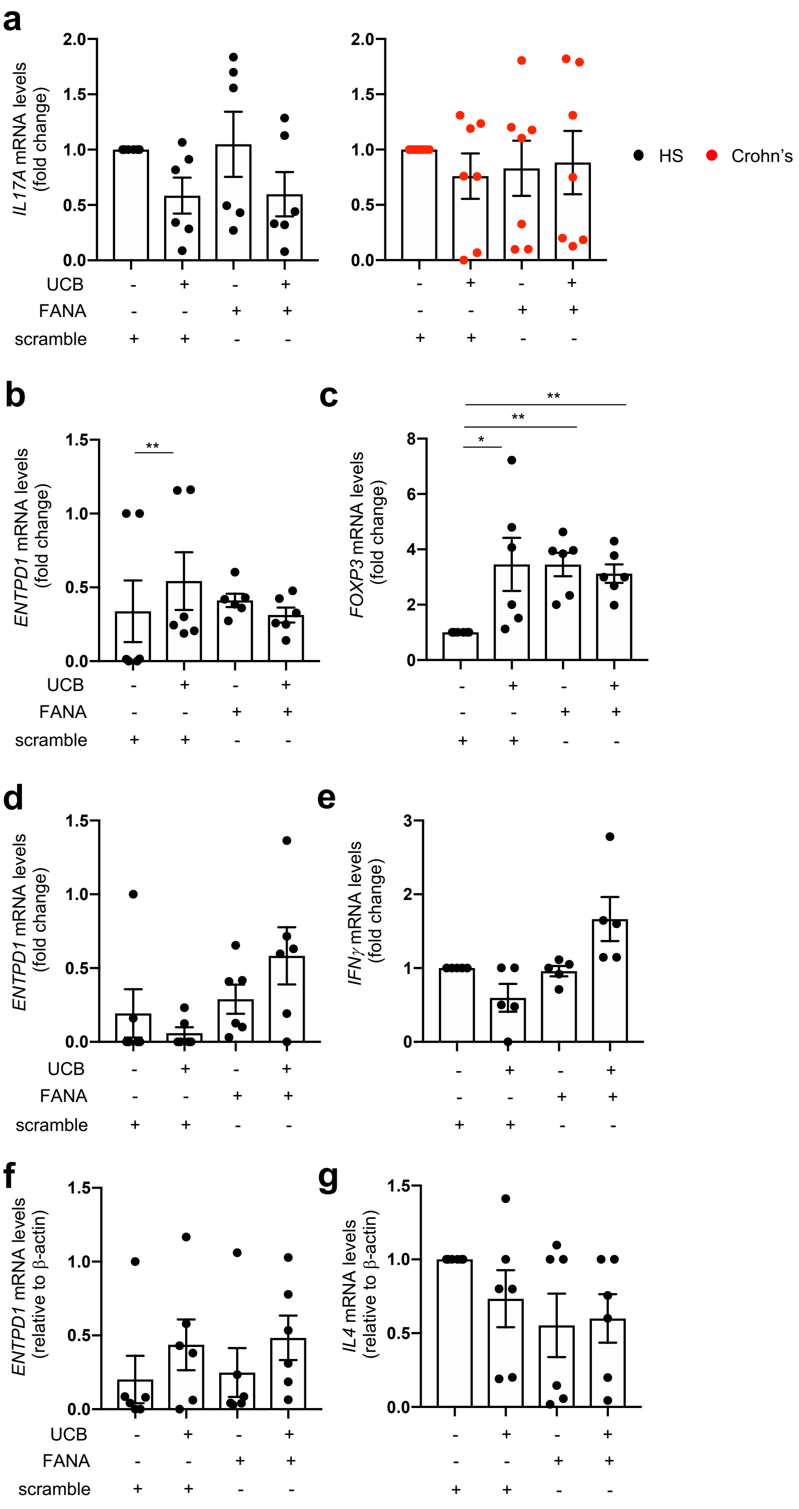

Supplementary Fig. 7. Effects of FANA oligonucleotide treatment on Th17, Treg, Th1 and Th2 cell gene expression.

Peripheral blood derived CD4 cells were polarized under Th17, Treg, Th1 and Th2 culture conditions for 5 days. Gene expression was assessed upon cell exposure to scramble, unconjugated

bilirubin (UCB), FANA-*PGK1* or FANA-*ALDOA* oligonucleotides or FANA oligonucleotides in combination with UCB. (a) Mean±SEM *IL-17A* mRNA levels in Th17 cells obtained from HS (n=6) and Crohn's disease patients (n=7). Mean±SEM (b) *ENTPD1* (encoding for CD39) and (c) *FOXP3* mRNA levels in Tregs from 6 HS. Mean±SEM (d) *ENTPD1* and (e) *IFNγ* mRNA levels in Th1 cells from HS (n=6 for *ENTPD1* and n=5 for *IFNγ*). Mean±SEM (f) *ENTPD1* and (g) *IL4* mRNA levels in Th2 lymphocytes from 6 HS. Results obtained in the presence of FANA-*PGK1* and FANA-*ALDOA* are pooled. *P≤0.05 and **P≤0.01 using ANOVA repeated measures followed by Tukey's multiple comparisons test.

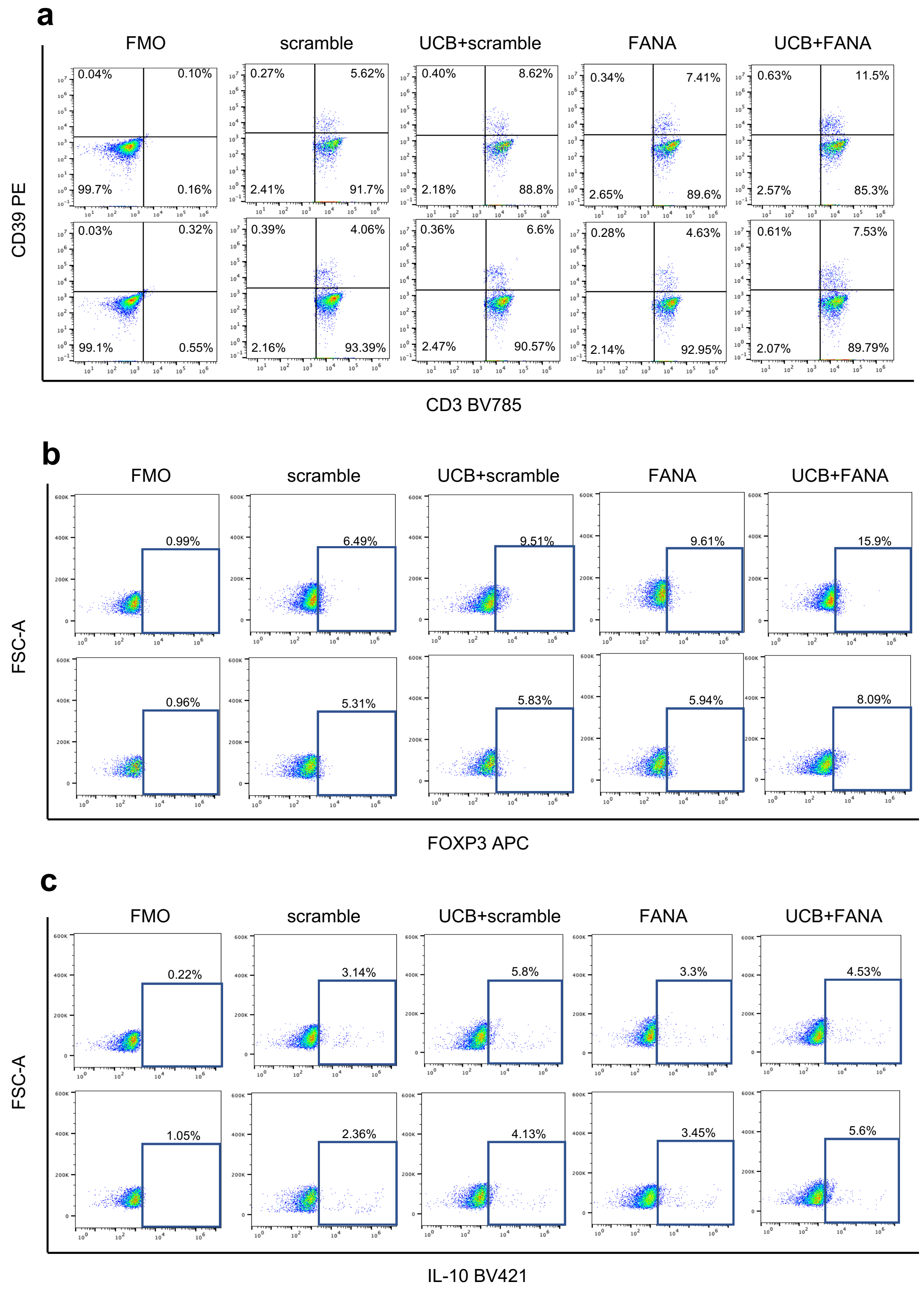

Supplementary Fig. 8. Silencing of *PGK1* or *ALDOA* boosts the immunoregulatory properties of unconjugated bilirubin in Th17 cells.

The frequency of $CD3^{+}CD39^{+}$ within IL-17A^{+} cells and that of FOXP3^{+} and IL-10^{+} cells within the T lymphocyte compartment was determined by flow cytometry after cell exposure to scramble,

unconjugated bilirubin (UCB), FANA oligonucleotides, or FANA oligonucleotides plus UCB. Cells were initially gated on live lymphocytes, single cells and subsequently on (a) IL-17-A$^+$ or (b-c) CD3 lymphocytes. (a) Pseudocolor plots of CD3 Brilliant Violet 785 (BV785) and CD39 PE fluorescence are shown in one representative healthy subject (HS) and one patient with Crohn's disease. Pseudocolor plots of forward scatter (FSC-A) and (b) FOXP3 APC or (c) IL-10 Brilliant Violet 421 (BV421) fluorescence are also shown. Cell frequencies are indicated in each quadrant. Quadrants and gates were drawn based on fluorescence minus one (FMO) staining control.

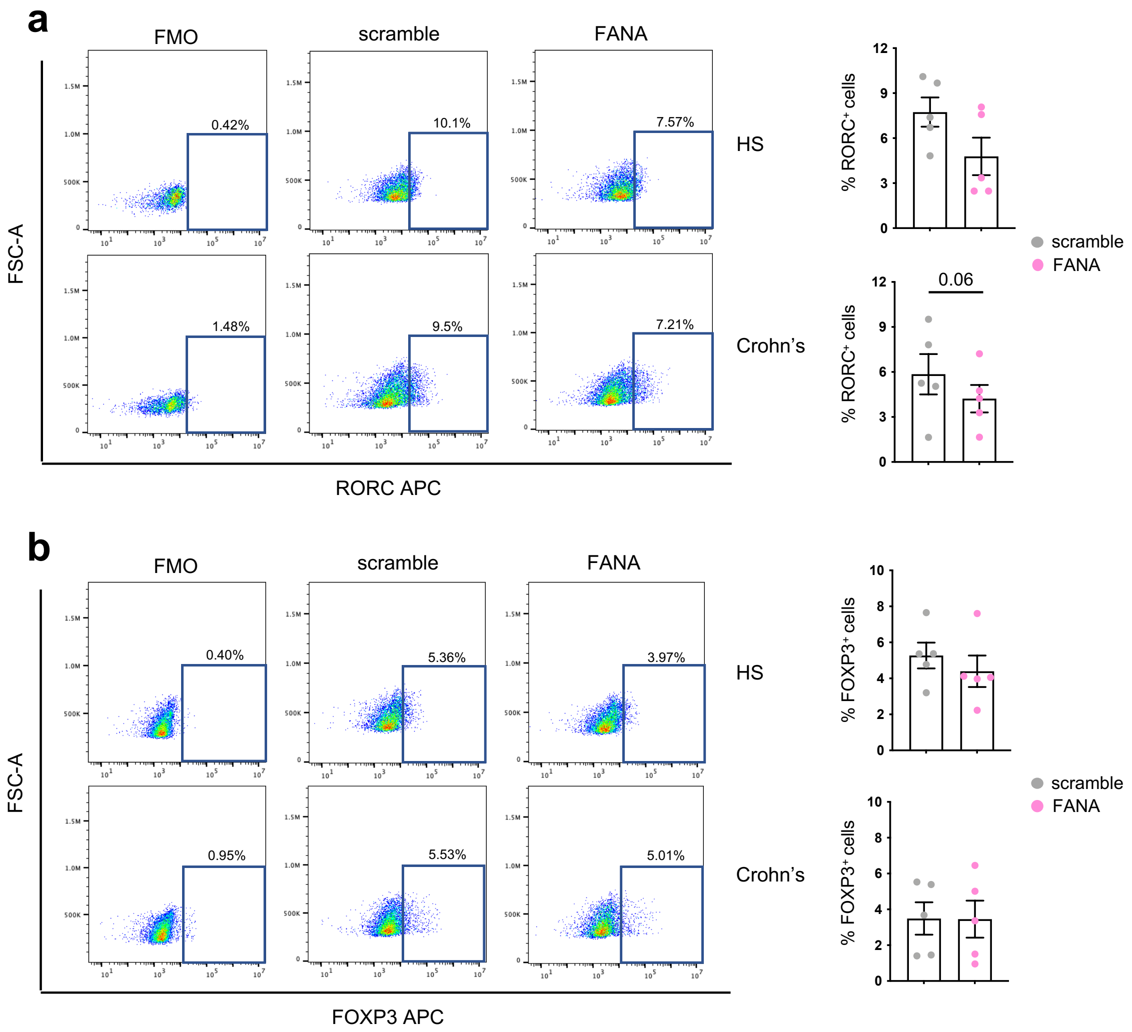

Supplementary Fig. 9. Effects of *PGK1* and *ALDOA* silencing on CD4 cell differentiation. Peripheral blood derived CD4 cells were polarized under Th17 culture conditions for 5 days. On day 2, FANA-*PGK1* or FANA-*ALDOA* were added to the cultures. Frequency of RORC$^+$ and FOXP3$^+$ cells within the CD4 cell compartment was measured at the end of polarization period by flow cytometry. (a-b) Pseudocolor plots of RORC APC or FOXP3 APC and FSC-A in untreated and FANA treated CD4 cells from one representative healthy subject (HS) and one patient with Crohn's disease are shown. Cell frequencies are indicated within the plots. Gates were drawn based on fluorescence minus one (FMO) staining control. Mean±SEM (a) RORC$^+$ and (b) FOXP3$^+$ lymphocytes with scramble and FANA treated CD4 cells of HS (n=5) and patients with Crohn's disease (n=5). Results obtained in the presence of FANA-*PGK1* and FANA-*ALDOA* are pooled. Comparisons were made using paired *t* test.

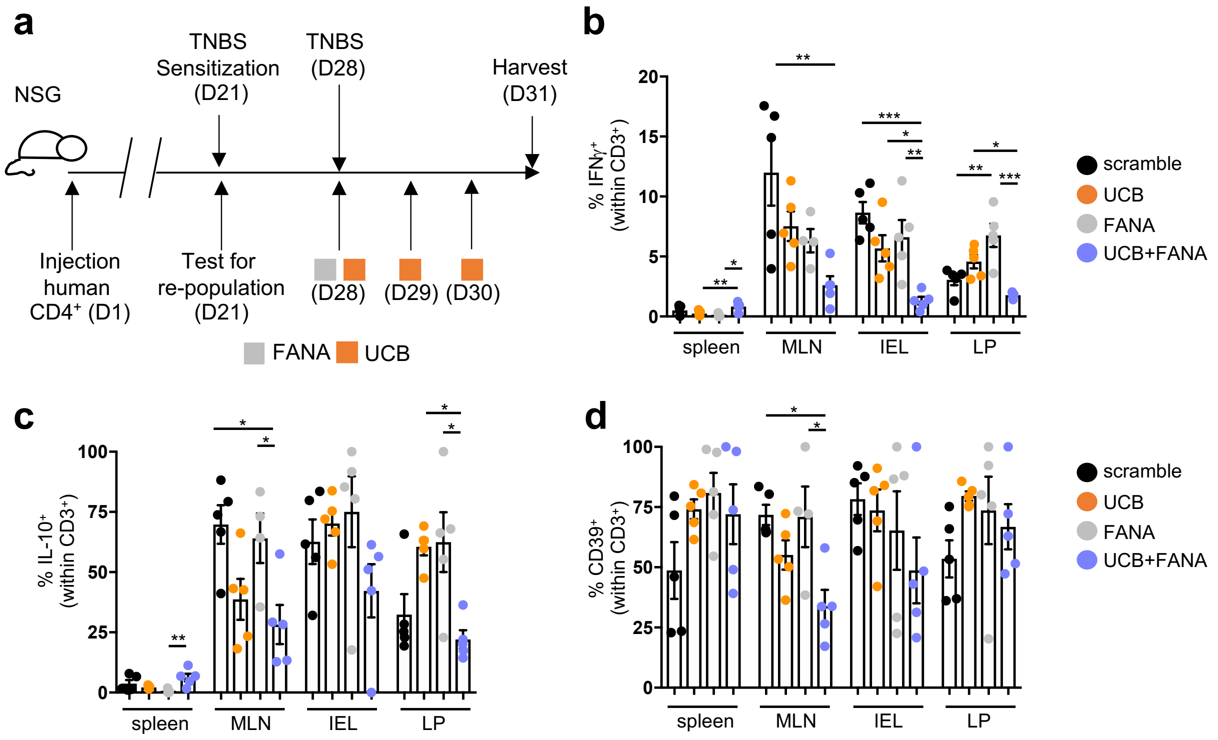

Supplementary Fig. 10. Effects of *PGK1* and *ALDOA* silencing on CD4 cell immune phenotype in TNBS induced colitis in *NOD/scid/gamma* mice.

(a) *NOD/scid/gamma* (NSG) female recipients were injected with *PGK1*$^+$ and *ALDOA*$^+$ CD4 cells, obtained from one healthy blood donor on day 1 (D1). After three weeks (day 21, D21), mice were checked for human chimerism. Mice showing more than 10% human chimerism were sensitized to TNBS and one week later (day 28, D28), administered a single enema of TNBS and a single intraperitoneal injection of scramble, unconjugated bilirubin (UCB), FANA-*PGK1* or FANA-*ALDOA* or a combination of UCB and FANA oligonucleotides. UCB administration was carried out once a day until harvest. After 72 hours (day 31, D31), mice were sacrificed, and organs harvested. The frequency of human IFNγ$^+$, IL-10$^+$ and CD39$^+$ cells within CD3 lymphocytes of spleen, mesenteric lymph node (MLN), intra-epithelial (IEL) and lamina propria (LP) derived lymphocytes was determined by flow cytometry. Cells were initially gated on live lymphocytes, then on single cells and subsequently on CD3 lymphocytes. Mean±SEM (b) IFNγ$^+$, (c) IL-10$^+$ and (d) CD39$^+$ cells in different compartments in scramble (n=5), UCB (n=5), FANA oligonucleotides (n=5) and FANA oligonucleotides plus UCB (n=5) treated mice. A combination of FANA oligonucleotides and UCB decreases the frequency of IFNγ$^+$ cells in MLN and IEL along with that of IL-10$^+$ and CD39$^+$ cells within the CD3 compartment in MLN. *P≤0.05, **P≤0.01 and *** P≤0.001 using one-way ANOVA followed by Tukey's multiple comparison test.

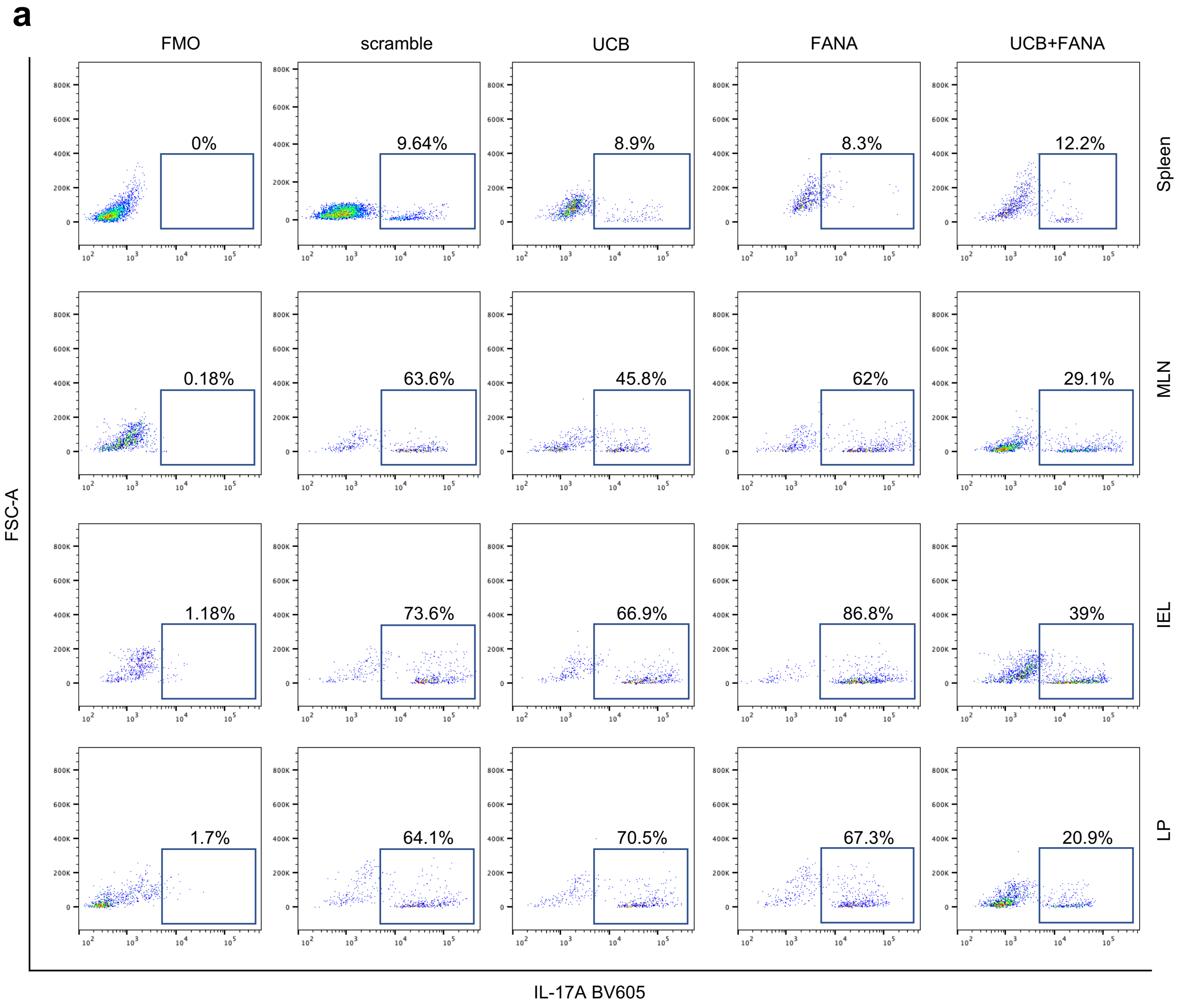
a
FMO
scramble
UCB
FANA
UCB+FANA
Spleen
MLN
IEL
LP
0%
9.64%
8.9%
8.3%
12.2%
0.18%
63.6%
45.8%
62%
29.1%
1.18%
73.6%
66.9%
86.8%
39%
1.7%
64.1%
70.5%
67.3%
20.9%
FSC-A
IL-17A BV605

b

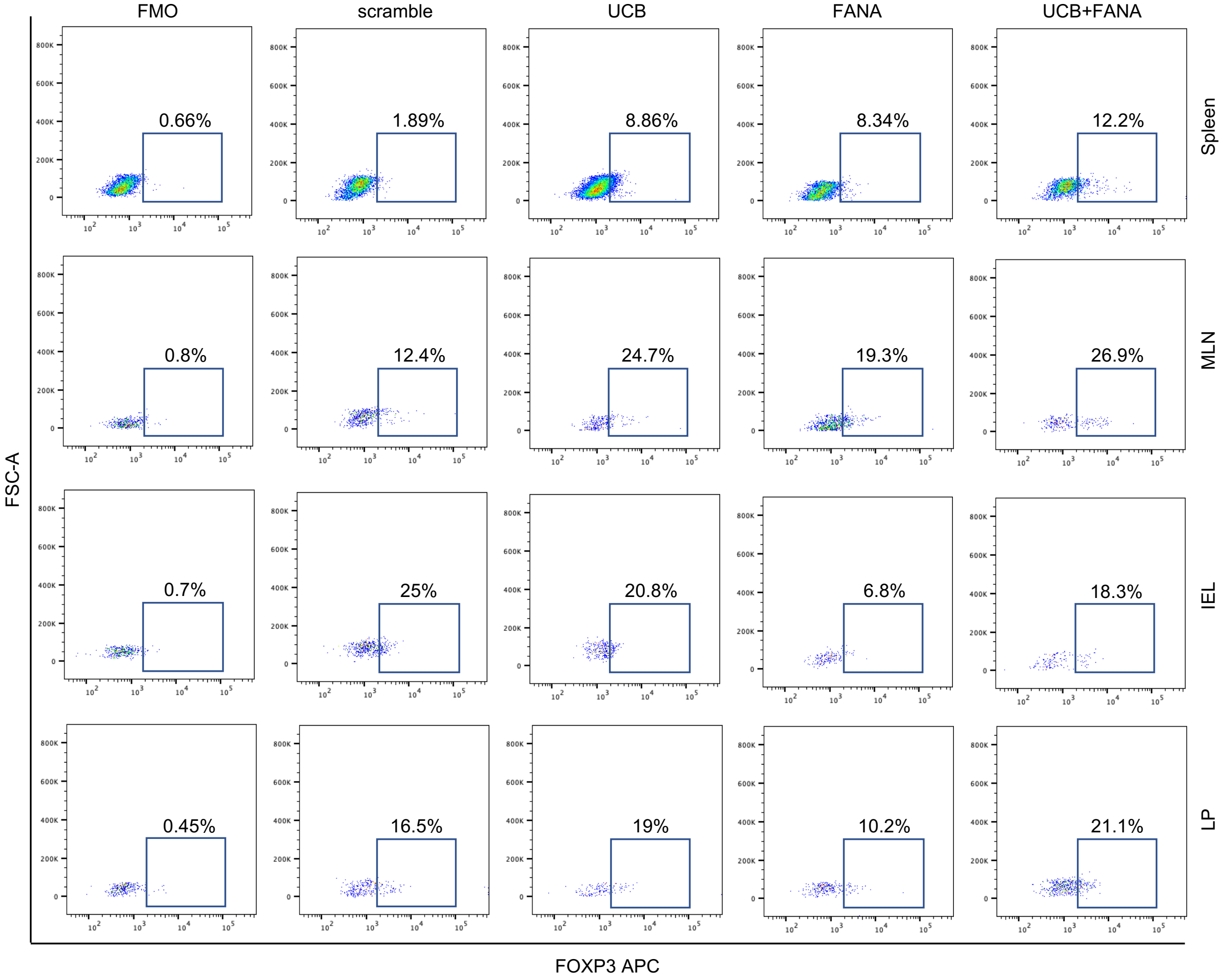

FMO
scramble
UCB
FANA
UCB+FANA
Spleen
MLN
IEL
LP
FSC-A
FOXP3 APC
0.66%
1.89%
8.86%
8.34%
12.2%
0.8%
12.4%
24.7%
19.3%
26.9%
0.7%
25%
20.8%
6.8%
18.3%
0.45%
16.5%
19%
10.2%
21.1%

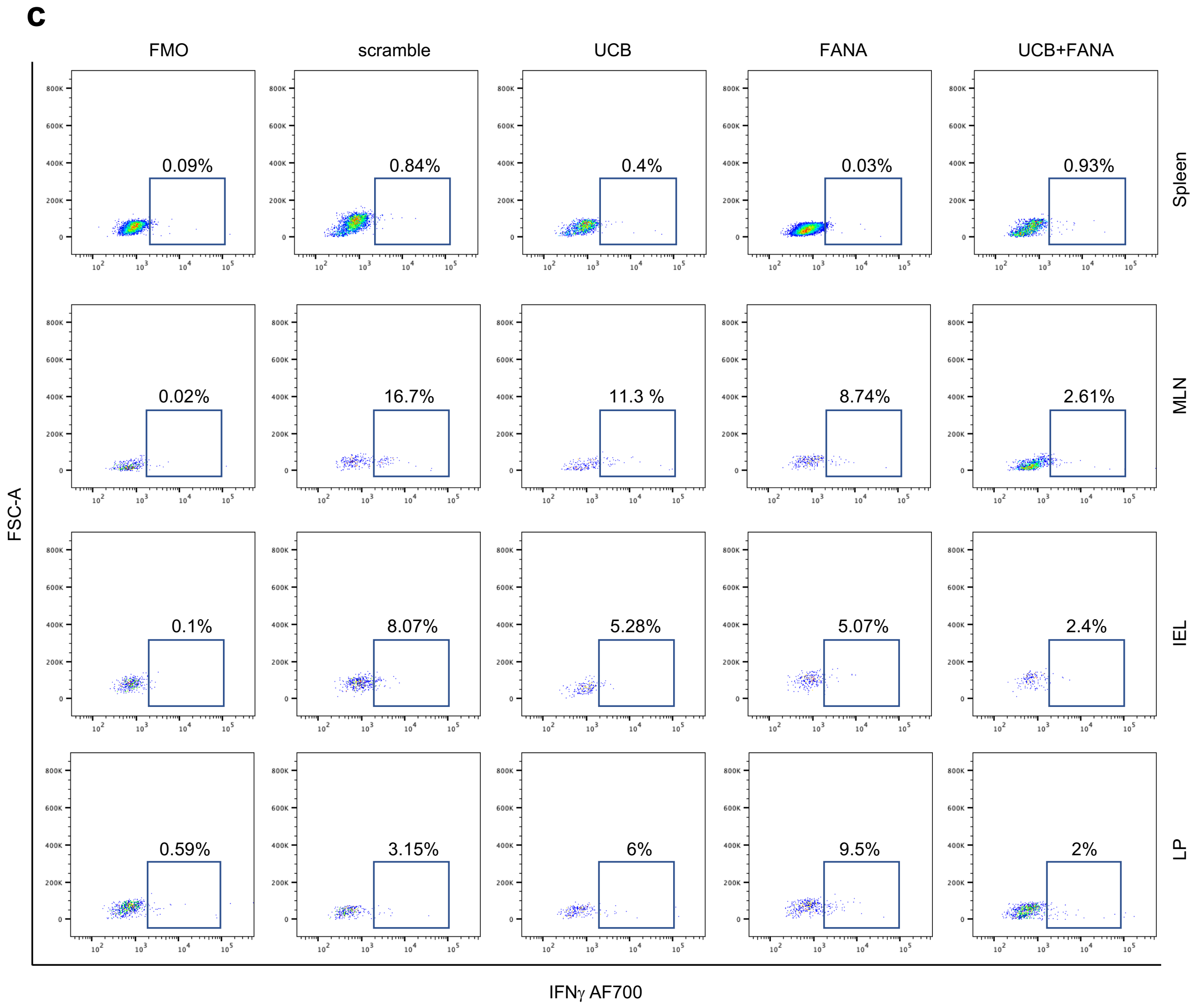
c
FMO
scramble
UCB
FANA
UCB+FANA
0.09%
0.84%
0.4%
0.03%
0.93%
Spleen
0.02%
16.7%
11.3 %
8.74%
2.61%
MLN
0.1%
8.07%
5.28%
5.07%
2.4%
IEL
0.59%
3.15%
6%
9.5%
2%
LP
FSC-A
IFNγ AF700

d

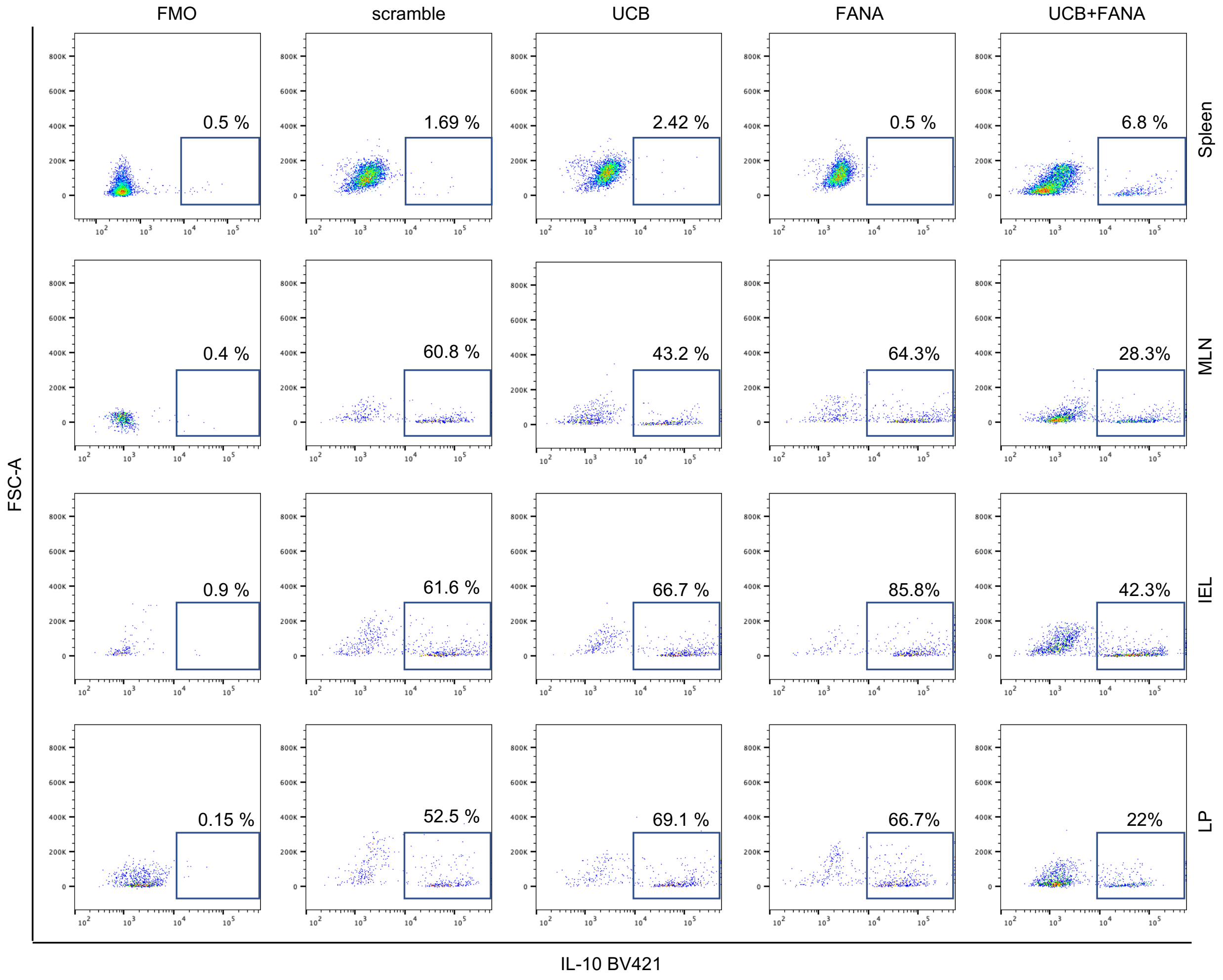

FMO
scramble
UCB
FANA
UCB+FANA
Spleen
MLN
IEL
LP
0.5 %
1.69 %
2.42 %
0.5 %
6.8 %
0.4 %
60.8 %
43.2 %
64.3%
28.3%
0.9 %
61.6 %
66.7 %
85.8%
42.3%
0.15 %
52.5 %
69.1 %
66.7%
22%
FSC-A
IL-10 BV421

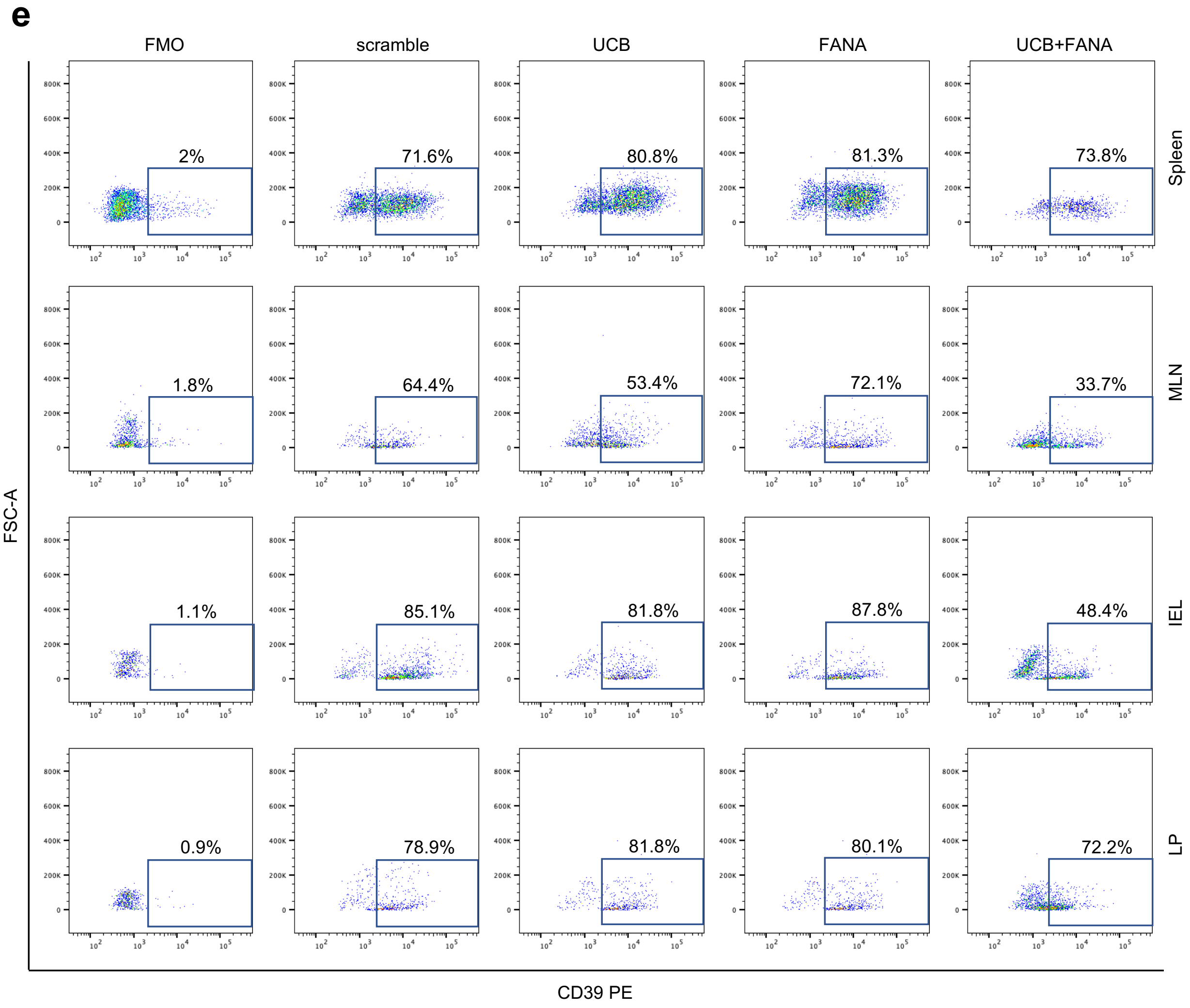
e
FMO
scramble
UCB
FANA
UCB+FANA
Spleen
MLN
IEL
LP
2%
71.6%
80.8%
81.3%
73.8%
1.8%
64.4%
53.4%
72.1%
33.7%
1.1%
85.1%
81.8%
87.8%
48.4%
0.9%
78.9%
81.8%
80.1%
72.2%
FSC-A
CD39 PE

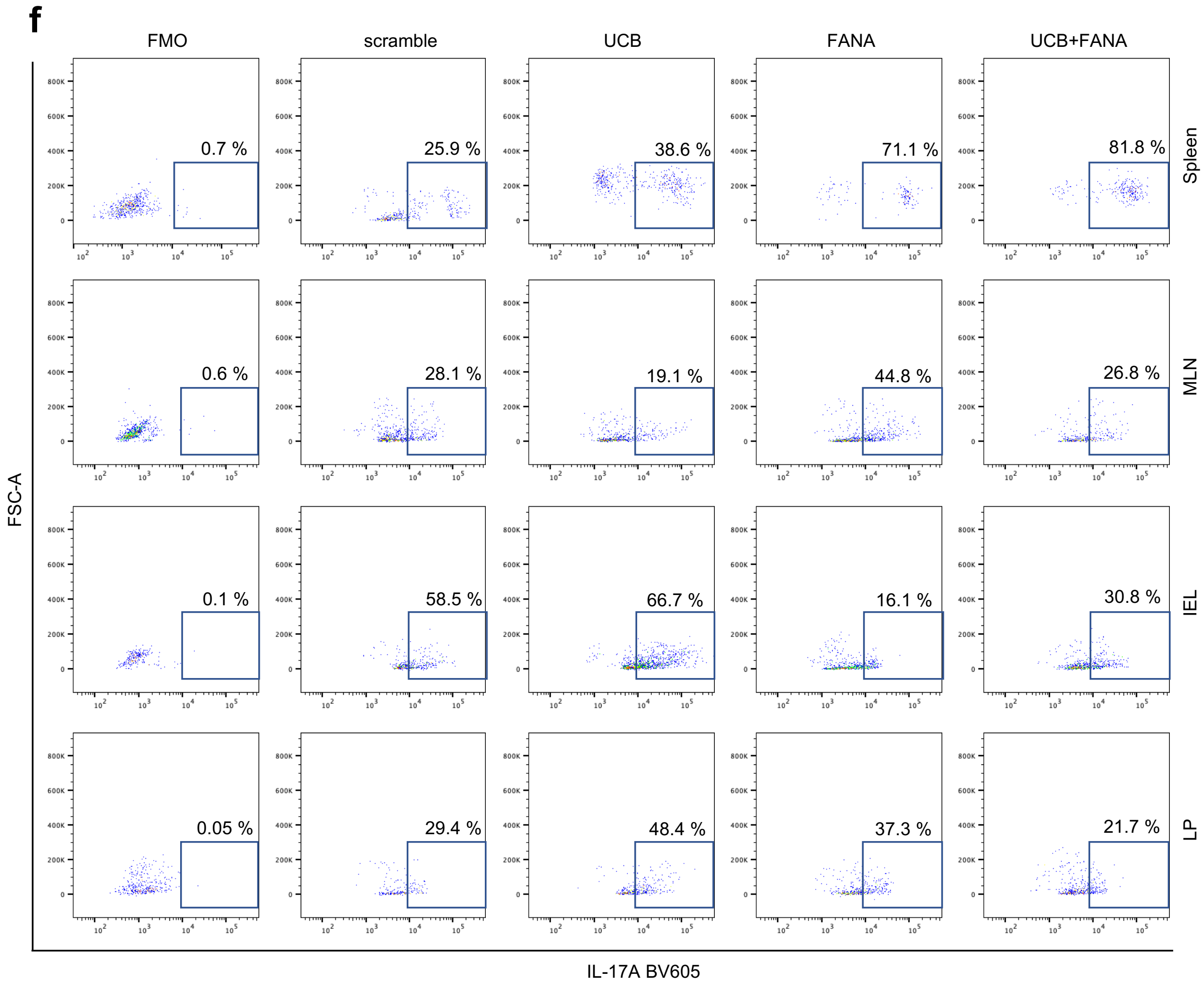
f
FMO
scramble
UCB
FANA
UCB+FANA
Spleen
MLN
IEL
LP
0.7 %
25.9 %
38.6 %
71.1 %
81.8 %
0.6 %
28.1 %
19.1 %
44.8 %
26.8 %
0.1 %
58.5 %
66.7 %
16.1 %
30.8 %
0.05 %
29.4 %
48.4 %
37.3 %
21.7 %
FSC-A
IL-17A BV605

g

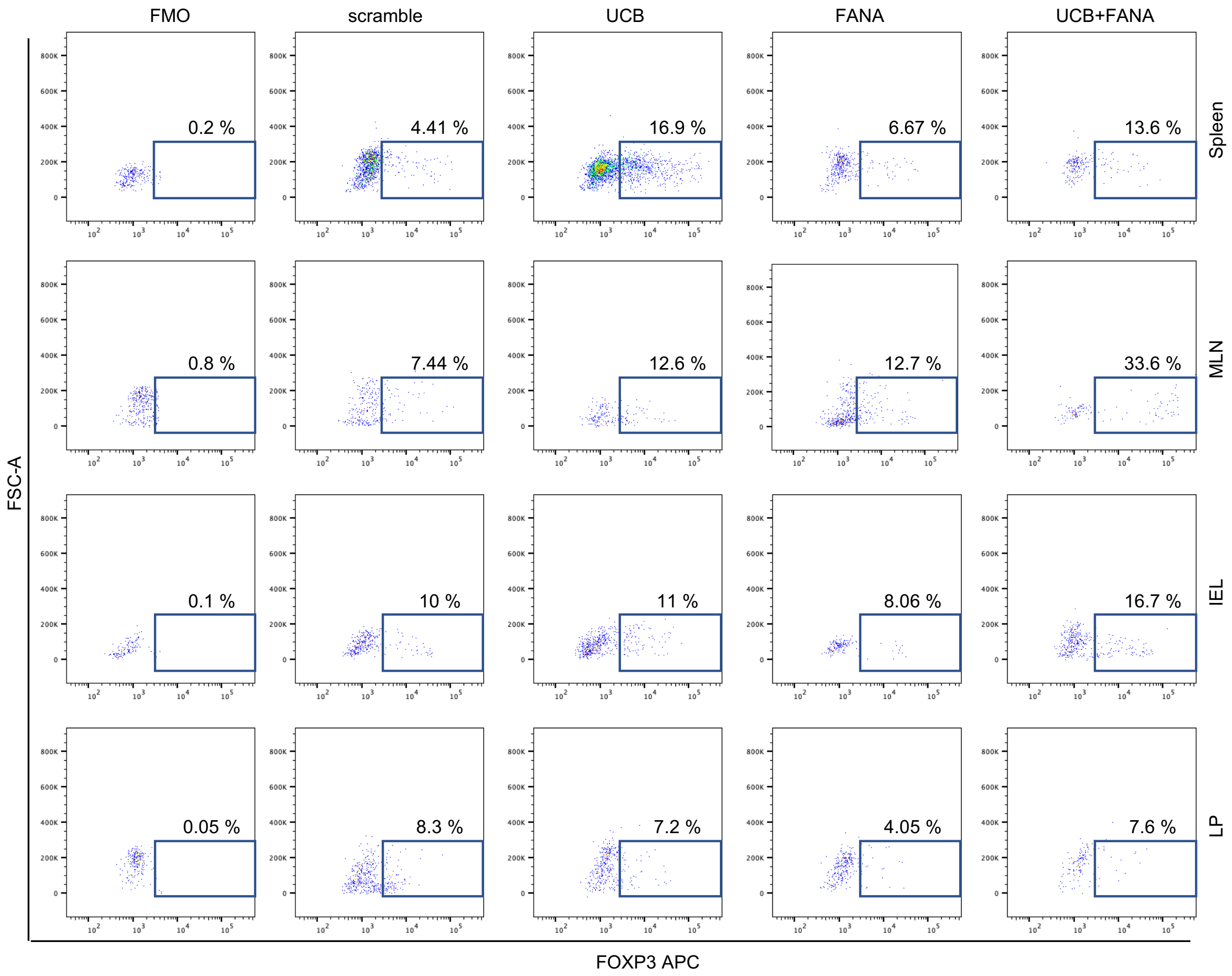

Supplementary Fig. 11. Effects of *PGK1* and *ALDOA* silencing on organ derived lymphocytes from humanized *NOD/scid/gamma* mice exposed to TNBS colitis.

Following reconstitution with healthy blood donor derived CD4 cells, *NOD/scid/gamma* mice were sensitized and administered TNBS. At the time of TNBS administration, some animals were treated with UCB, FANA oligonucleotides specifically silencing *PGK1* or *ALDOA*, or with a combination of FANA oligonucleotides and UCB. Mice were harvested 72 hours later and frequency of human IL-17A$^+$, FOXP3$^+$, IFNγ$^+$, IL-10$^+$, CD39$^+$ within the CD3$^+$ compartment, and of IL-17A$^+$ and FOXP3$^+$ cells within the CD3$^+$CD39$^+$ subset of spleen, mesenteric lymph node (MLN), intra-epithelial (IEL) and lamina propria (LP) derived lymphocytes was determined by flow cytometry. Cells were initially gated on live lymphocytes, then on single cells and subsequently gated on CD3$^+$ (a-e) or CD3$^+$CD39$^+$ (f-g) lymphocytes. Cell frequencies are indicated next to the gate or within each quadrant. Representative FSC-A and (a) IL-17A Brilliant Violet 605 (BV605), (b) FOXP3 APC, (c) IFNγ Alexa Fluor 700 (AF700), (d) IL-10 Brilliant Violet 421 (BV421) and (e) CD39 PE flow cytometry plots of CD3$^+$ lymphocytes in the spleen, MLN, IEL and LP of a representative mouse treated with scramble, UCB, FANA oligonucleotide or FANA oligonucleotide plus UCB are shown. Pseudocolor plots of FSCA-A and (f) IL-17A Brilliant Violet 605 or (g) FOXP3 APC fluorescence within CD3$^+$CD39$^+$ cells are also shown.

Supplementary Table 1. Demographic and clinical data of Crohn's disease patients.

Crohn's Disease (n=71)	
Active/Inactive	29/42
Sex (F/M)	45/26
Montreal Age*	'A1' n=12<br>'A2' n=49<br>'A3' n=6
Montreal Type*	'B1' n=33<br>'B2' n=17<br>'B3' n=18
Infliximab	29
Adalimumab	1
Steroids	6
Mercaptopurine	12
Ustekinumab	12
Vedolizumab	6

F: female
M: male
Montreal Age: 'A1' < 16 years; 'A2' 17-40 years; 'A3' > 40 years; * information about Montreal age was not available for 4 patients.
Montreal Type: 'B1' non-stricturing, non-penetrating; 'B2' stricturing; 'B3' penetrating; * information about Montreal type was not available for 3 patients.
