# Supplementary material for: Blockade of PGK1 and ALDOA enhances bilirubin control of Th17 cells in Crohn’s disease
Source: Commun Biol. 2022 Sep 21;5:994. doi: 10.1038/s42003-022-03913-9 (PMC9492699; doi:10.1038/s42003-022-03913-9)
Supplement: Supplementary file 3 — Description of Additional Supplementary Files [file 42003_2022_3913_MOESM3_ESM.pdf]

Description of Additional Supplementary Files

File name: Supplementary Data 1

Description: The source data behind the graphs presented in the main figures.
